# Subgroup Differences in Parenting Stress and Life Satisfaction Among Parents of Children with Disabilities Receiving Adapted Physical Activity Services

**DOI:** 10.3390/healthcare14111434

**Published:** 2026-05-22

**Authors:** Jinwoo Park, Seunghyun Jang

**Affiliations:** 1 Department of Physical Education, Pusan National University, Busan 46241, Republic of Korea; wave3305@pusan.ac.kr; 2 Department of Sport and Leisure Studies, Keimyung University, Daegu 42601, Republic of Korea

**Keywords:** parenting stress, life satisfaction, parents of children with disabilities, adapted physical activity, family-centered care, mental health, developmental rehabilitation services, subgroup differences

## Abstract

**Highlights:**

**What are the main findings?**

- Female parents reported higher social and psychological stress than male parents, and household income and disability severity were associated with several domains of parenting stress and life satisfaction.
- Household income showed the largest and most robust association with economic stress in the reported analyses.
- Disability severity was associated with several stress domains and life satisfaction, whereas its association with economic stress emerged only after covariate adjustment and should be interpreted as adjustment-sensitive.

**What is the implication of the main finding?**

- The findings may help identify parent subgroups who could benefit from additional family-centered support within developmental rehabilitation service contexts, without implying that APA services themselves caused these patterns.
- Because the study was cross-sectional and based on self-reported data, longitudinal, comparative, and service-level studies are needed before drawing conclusions about causal effects or cumulative service-related burden.

**Abstract:**

Background/Objectives: Parenting stress and life satisfaction are important indicators of family well-being and parent mental health in families of children with disabilities. However, limited empirical attention has been given to how these outcomes differ among parents whose children receive adapted physical activity (APA) services within South Korea’s Developmental Rehabilitation Service system. This cross-sectional study examined subgroup differences in parenting stress and life satisfaction according to sociodemographic, disability-related, and service-utilization characteristics among parents of children receiving APA services. Methods: Data were collected from 295 parents of school-aged children with disabilities enrolled in APA services at child development centers. Welch-type tests, Welch’s ANOVA or one-way ANOVA, Pearson correlation analyses, Benjamini–Hochberg FDR adjustment, and supplementary analyses of covariance (ANCOVA) were used to examine group differences and the stability of selected associations after adjustment for prespecified covariates. Confirmatory factor analysis and gender-based measurement invariance testing were also conducted for the adapted parenting stress scale. Results: Parenting stress subdomains were positively correlated with one another (r = 0.19–0.53) and negatively correlated with life satisfaction (r = −0.28 to −0.40). Female parents reported higher social and psychological stress than male parents. Household income showed the largest association with economic stress, and significant differences were also observed according to parental age, education level, disability severity, and selected service-utilization characteristics. Some associations remained after ANCOVA adjustment, whereas others were attenuated or emerged only after adjustment. Conclusions: The findings indicate subgroup differences in parenting stress and life satisfaction among parents of children receiving APA services. Because the study used a cross-sectional, self-reported design with convenience sampling and an adapted instrument, the results should be interpreted as preliminary associative evidence rather than evidence of causal or service-specific effects. Future longitudinal, comparative, and service-level research is needed to clarify how APA service contexts relate to caregiver well-being over time.

## 1. Introduction

Parenting a child with a disability often involves sustained caregiving responsibilities that extend beyond routine childrearing and may be associated with parental stress, reduced life satisfaction, and broader family well-being challenges [1,2,3,4,5,6,7,8,9,10]. Recent evidence also suggests that caregiver mental health concerns are not isolated clinical issues but public health concerns. For example, Soh et al. reported median prevalence estimates of 33.35% for depression, 35.25% for anxiety, and 49.26% for caregiver burden among informal caregivers in an umbrella review of meta-analyses [11]. These estimates reinforce the importance of examining caregiver stress and life satisfaction as health-related outcomes within disability service contexts.

Parenting stress in families of children with disabilities is multidimensional and may include economic burden, psychological strain, physical fatigue, and social restriction [8,9]. These domains are closely associated with parent mental health, life satisfaction, and family quality of life [2,9,10]. However, caregiving burden is not experienced uniformly across families. It may differ according to parental resources, child disability characteristics, and the practical conditions under which services are accessed, financed, and sustained.

In South Korea, the Developmental Rehabilitation Service (DRS) system supports children with disabilities through structured interventions such as language therapy, sensory-motor training, and adapted physical activity (APA). APA services delivered through child development centers emphasize motor development, physical fitness, and structured movement experiences. Previous studies have mainly focused on child outcomes, including motor proficiency, participation, and physical or psychosocial development [12,13,14]. Comparatively less is known about parenting stress and life satisfaction among parents whose children participate in APA-based services.

This study uses Lazarus and Folkman’s transactional model of stress and coping as a heuristic framework rather than as a fully tested explanatory model [15]. The model helps interpret how caregiving demands and available resources may be related to parental stress. However, because this study did not directly measure appraisal processes, coping strategies, caregiving hours, or informal support, the model is used to guide interpretation rather than to establish a formal causal mechanism.

APA services may provide a relevant context for examining caregiver well-being because continued participation can involve scheduling, transportation, communication with providers, and family support for activity participation. Nevertheless, the present study does not include a comparison group from other rehabilitation modalities; therefore, it does not test whether APA is uniquely or causally different from other service contexts. Any distinctiveness of APA as a family care context should be regarded as a hypothesis for future comparative research.

Despite growing implementation of APA within developmental rehabilitation services, limited research has examined how parenting stress and life satisfaction vary across subgroups of parents receiving these services. Identifying such subgroup differences can provide preliminary information for caregiver screening, family-centered service planning, and future longitudinal or comparative studies.

Accordingly, this exploratory cross-sectional study examined whether parenting stress and life satisfaction differed according to sociodemographic, disability-related, and service-utilization characteristics among parents of school-aged children with disabilities receiving APA services. The study was not designed to test causal effects of APA participation, but to describe within-population variation among APA-receiving families.

This study addresses the following research questions:

(1) Do parenting stress and life satisfaction differ according to parents’ sociodemographic characteristics among parents whose children receive APA services?

(2) Do parenting stress and life satisfaction differ according to children’s disability characteristics and service utilization patterns among parents whose children receive APA services?

## 2. Materials and Methods

### 2.1. Participants

This study used a cross-sectional survey design. Participants were parents of school-aged children with disabilities who were receiving adapted physical activity services at child development centers in South Korea. These services were delivered as part of the Developmental Rehabilitation Service system and included structured physical activity interventions designed to promote motor development, physical fitness, and social interaction among children with disabilities.

Participants were recruited using convenience sampling from 16 child development centers located in Busan, Ulsan, Gyeongnam, and Jeju, South Korea, all of which provided APA-based developmental rehabilitation services. Centers were selected based on their provision of APA services and willingness to cooperate with the study. Data were collected over a two-month period starting in September 2024. Approximately 400 parents were approached for participation, and 320 parents initially provided survey responses, yielding an approximate response rate of 80.0%. After excluding 25 questionnaires due to incomplete responses, patterned responses, or failure to meet the inclusion criteria, 295 valid cases were included in the final analysis. Missing data were handled using listwise deletion, and only cases with complete responses on the study variables were retained for analysis. The valid response rate among returned questionnaires was 92.2%.

Among the participants, 34.2% were fathers (n = 101) and 65.8% were mothers (n = 194). Regarding age distribution, 11.9% were in their 30s (n = 35), 59.0% were in their 40s (n = 174), and 29.2% were aged 50 years or older (n = 86). In terms of educational attainment, 15.3% had completed high school or less (n = 45), 80.0% had a university degree (n = 236), and 4.7% had completed graduate school (n = 14).

With respect to monthly household income, 4.7% reported USD 1500–2300 (n = 14), 22.4% reported USD 2300–3100 (n = 66), 47.5% reported USD 3100–3900 (n = 140), and 25.4% reported USD 3900 or above (n = 75).

Regarding children’s disability characteristics, autism spectrum disorder was the most prevalent diagnosis (53.9%, n = 159), followed by intellectual disability (36.9%, n = 109), emotional/behavioral disorder (5.1%, n = 15), and physical disability (4.1%, n = 12). In terms of disability severity, 38.3% of children were classified as having mild disabilities (n = 113), 50.5% as moderate disabilities (n = 149), and 11.2% as severe disabilities (n = 33).

This study followed the Strengthening the Reporting of Observational Studies in Epidemiology (STROBE) guidelines. The study was conducted in accordance with the Declaration of Helsinki and approved by the Institutional Review Board of Silla University (approval code: 1041449-202404-HR-001; approval date: 2 May 2024). All participants were informed about the purpose and procedures of the study, and written informed consent was obtained prior to participation. Participation was voluntary, and respondents were assured of the confidentiality and anonymity of their responses. The completed STROBE checklist is provided as Supplementary Material.

Inclusion criteria required that participants be parents or legal guardians of school-aged children with registered disabilities who were actively receiving APA services within a child development center at the time of data collection. Participants were excluded if their child had been receiving the service for less than one month or if they were unable to complete the questionnaire independently. The participant characteristics are summarized in Table 1.

### 2.2. Service Utilization Characteristics

Information on service utilization was collected to examine group differences in parenting stress and life satisfaction according to patterns of participation in adapted physical activity services delivered within developmental rehabilitation services. For group comparison analyses, two service-related variables were examined: duration of participation and monthly out-of-pocket treatment cost.

Duration of participation was categorized into five groups: less than 6 months (2.0%, n = 6), 6–12 months (9.8%, n = 29), 1–2 years (22.4%, n = 66), 2–3 years (30.2%, n = 89), and 3 years or more (35.6%, n = 105). These cut-off points were determined based on the observed distribution of participation durations in the current sample and reflect meaningful stages of service engagement within the DRS framework, ranging from initial service uptake (less than 6 months) to established long-term participation (3 years or more), consistent with the service renewal and eligibility assessment cycles of the Developmental Rehabilitation Service program [16]. The largest proportion of participants had engaged in services for three years or longer, followed by those participating for two to three years.

Monthly treatment cost referred to the parent-reported out-of-pocket payment for APA-based developmental rehabilitation services after excluding government voucher support. The variable did not include transportation expenses, other rehabilitation services, medical costs, educational expenses, or total disability-related care expenses. Costs were converted from Korean won to U.S. dollars (USD) for analysis. Costs were categorized into five groups: less than USD 80 (7.8%, n = 23), USD 80–115 (23.4%, n = 69), USD 115–155 (21.0%, n = 62), USD 155–195 (23.7%, n = 70), and USD 195 or above (24.1%, n = 71). These categories were informed by the observed distribution of out-of-pocket costs in the sample and by policy-relevant benchmarks under the DRS program. As of 2024, the standard government voucher unit price was approximately USD 22 per session, whereas the average market service price was approximately USD 40 per session, creating potential variation in out-of-pocket expenses across families [16]. Because the cut points were partly data-driven, findings involving treatment cost should be interpreted as exploratory. The service utilization characteristics of the participants are summarized in Table 2.

### 2.3. Instruments

#### 2.3.1. Parenting Stress

Parenting stress refers to the daily stress experienced by parents in the process of raising their children and arises when parenting demands exceed available coping resources [15,17,18]. In families of children with disabilities, parenting stress may be intensified due to additional caregiving demands, behavioral challenges, and financial pressures.

In this study, parenting stress was measured using a modified version of the Parenting Stress Index (PSI) originally developed by Abidin [17]. The instrument was adapted for parents of children with disabilities based on prior Korean studies [19,20,21]. The final scale consisted of 14 items across four subdomains: economic stress (4 items), psychological stress (3 items), physical stress (4 items), and social stress (3 items).

The adaptation process involved several modifications to the original PSI items. Specifically, items that were deemed culturally inappropriate or less relevant to the Korean disability service context were reviewed and reworded based on expert consultation with adapted physical activity specialists and review of prior Korean studies utilizing similar instruments [20,21]. Items were modified to more accurately reflect the specific caregiving experiences of parents of children with disabilities participating in structured rehabilitation services, particularly with respect to economic and social stressors. No formal pilot study or independent cross-cultural validation was conducted; however, the adapted scale demonstrated adequate psychometric properties in the current sample, as evidenced by the factor structure and reliability coefficients reported below. Future studies should consider formal cross-cultural validation of this instrument to strengthen its applicability across diverse samples.

All items were rated on a five-point Likert scale ranging from 1 (strongly disagree) to 5 (strongly agree), with higher scores indicating higher levels of parenting stress.

Exploratory factor analysis using principal component analysis initially supported the four-factor structure. Because the parenting stress subdomains were theoretically and empirically correlated, an oblique rotation sensitivity check was additionally conducted. The oblique solution retained the same four-factor pattern, and the substantive interpretation of economic, psychological, physical, and social stress remained unchanged. The Kaiser-Meyer-Olkin (KMO) measure was 0.82, and Bartlett’s test of sphericity was significant (χ^2^ = 2218.71, *p* < 0.001). The four factors accounted for 72.92% of the total variance. Cronbach’s α and McDonald’s ω coefficients indicated acceptable to good internal consistency: economic stress (α = 0.85, ω = 0.89), psychological stress (α = 0.77, ω = 0.79), physical stress (α = 0.83, ω = 0.90), and social stress (α = 0.80, ω = 0.82). The reliability of psychological stress was acceptable but comparatively lower and should therefore be interpreted cautiously.

A confirmatory factor analysis was conducted to further examine the four-factor structure of the adapted parenting stress scale. The hypothesized four-factor model showed acceptable fit to the data, χ^2^(71) = 142.36, χ^2^/df = 2.01, CFI = 0.933, TLI = 0.929, RMSEA = 0.058, and SRMR = 0.046. Model fit was interpreted using commonly applied acceptable-fit criteria, including CFI and TLI values of approximately 0.90 or higher and RMSEA and SRMR values below conventional acceptable thresholds. We acknowledge that these values do not meet stricter cutoffs such as CFI/TLI ≥ 0.95. These results provided additional support for the four-factor structure consisting of economic, physical, psychological, and social stress.

Gender-based measurement invariance testing was also conducted because gender differences in social and psychological stress were among the main findings. The configural model showed acceptable fit, χ^2^(142) = 223.48, CFI = 0.938, TLI = 0.922, RMSEA = 0.062, and SRMR = 0.053, indicating that the four-factor structure was comparable across male and female parents under the same acceptable-fit convention described above. Metric invariance was also supported, χ^2^(152) = 236.91, CFI = 0.936, TLI = 0.925, RMSEA = 0.061, and SRMR = 0.058, with changes in CFI and RMSEA within recommended thresholds (ΔCFI = −0.002; ΔRMSEA = −0.001). Scalar invariance was additionally supported, χ^2^(162) = 253.27, CFI = 0.932, TLI = 0.924, RMSEA = 0.062, and SRMR = 0.060, with acceptable changes in fit indices (ΔCFI = −0.004; ΔRMSEA = 0.001). These results suggest that the adapted parenting stress scale functioned comparably across gender groups, supporting cautious interpretation of gender-related subgroup differences. However, because the scale was adapted and tested within the same sample used for subgroup inference, further validation in independent samples remains warranted. Detailed results of the CFA and gender-based measurement invariance testing are provided in Supplementary Table S1.

#### 2.3.2. Life Satisfaction

Life satisfaction refers to an individual’s overall cognitive evaluation of the quality of their life based on personal expectations, values, and achievements [22].

In this study, life satisfaction was measured using a five-item scale adapted from the general quality-of-life measure developed by Rodgers and Converse [23] and the Satisfaction with Life Scale proposed by Diener et al. [22], with additional modifications based on Jeong and Jeong [24] to reflect the experiences of parents raising children with disabilities.

All items were rated on a five-point Likert scale ranging from 1 (strongly disagree) to 5 (strongly agree), with higher scores indicating greater life satisfaction.

Exploratory factor analysis confirmed a single-factor structure. The Kaiser-Meyer-Olkin (KMO) value was 0.86, and Bartlett’s test of sphericity was significant (χ^2^ = 797.87, *p* < 0.001). The factor explained 69.01% of the total variance. The internal consistency reliability coefficients (Cronbach’s α = 0.87; McDonald’s ω = 0.92) indicated good reliability. 

The factor loadings and reliability coefficients for the parenting stress and life satisfaction measures are presented in Table 3.

### 2.4. Data Analysis

All statistical analyses were conducted using IBM SPSS Statistics (Version 29; IBM Corp., Armonk, NY, USA). Prior to the main analyses, assumptions of normality were evaluated using skewness and kurtosis indices, and homogeneity of variance was assessed using Levene’s test. Descriptive statistics, including frequencies, percentages, means, standard deviations, standard errors, and 95% confidence intervals where appropriate, were used to summarize participant characteristics and outcome distributions.

Pearson correlation analysis was conducted to examine relationships among parenting stress subdomains and life satisfaction. Because all data were based on parent self-report, the possibility of common method bias and subjective reporting bias was considered when interpreting the findings. Although the parenting stress subdomains were moderately intercorrelated, univariate Welch-type tests were retained because the study aimed to describe outcome-specific subgroup differences rather than estimate a single multivariate group effect, and because several subgroup sizes were small and unequal.

To examine group differences in parenting stress and life satisfaction according to demographic and service-utilization characteristics, variance-robust Welch-type tests were conducted for variables with two categories. For variables with three or more categories, Welch’s ANOVA was performed when the assumption of homogeneity of variance was violated; otherwise, one-way ANOVA was used. In cases of significant omnibus effects, post hoc comparisons were conducted using the Games-Howell procedure when equal variances or group sizes could not be assumed.

The primary analytic approach was group comparison rather than regression-based prediction because the study was exploratory and descriptive in purpose. Accordingly, terms such as “predictor” were avoided unless referring to supplementary adjusted models, and the findings were interpreted as subgroup differences or associations rather than causal effects.

To address the risk of Type I error inflation due to multiple subgroup comparisons, Benjamini-Hochberg false discovery rate (FDR) adjustment was applied within each outcome family. Raw *p*-values and FDR-adjusted *p*-values were reported together. Effect sizes were reported using η^2^ with bootstrapped 95% confidence intervals, and ω^2^ was additionally reported as a sensitivity estimate for omnibus Welch-type tests. Because Welch’s ANOVA does not partition variance in the same manner as conventional ANOVA, η^2^ and ω^2^ were interpreted cautiously as approximate effect-size estimates. Findings that disappeared after covariate adjustment or emerged only after covariate adjustment were interpreted as adjustment-sensitive because they may reflect statistical dependency on the included covariates rather than stable subgroup effects. Supplementary ANCOVA analyses were conducted to examine whether selected group differences remained stable after controlling for conceptually relevant covariates. Household income, education level, disability severity, and duration of service participation were selected as covariates because prior research and the present conceptual framework identify these variables as potential confounders of parental stress and life satisfaction. When a variable served as the fixed factor, it was not simultaneously entered as a covariate. Multicollinearity among covariates was assessed using tolerance and variance inflation factor (VIF) values. The homogeneity of regression slopes assumption was examined by testing fixed factor × covariate interaction terms for each ANCOVA model. No serious multicollinearity was detected, with tolerance values ranging from 0.74 to 0.93 and VIF values ranging from 1.08 to 1.35. The homogeneity of regression slopes assumption was generally satisfied across the main ANCOVA models; therefore, the adjusted analyses were considered appropriate.

## 3. Results

Unless otherwise noted, the results below are presented as exploratory subgroup differences and associations. Interpretive statements implying causal, longitudinal, or service-specific effects were avoided because of the cross-sectional self-report design. Small subgroup findings, particularly graduate education (n = 14), physical disability (n = 12), emotional/behavioral disorder (n = 15), and severe disability (n = 33), should be interpreted cautiously. Detailed subgroup descriptive statistics, including sample sizes, means, standard deviations, standard errors, and 95% confidence intervals for each outcome, are provided in Supplementary Table S3.

### 3.1. Correlation Analysis

Pearson correlation analysis showed that all subdomains of parenting stress were significantly and positively correlated with one another (r = 0.19–0.53, *p* < 0.01). Social stress demonstrated the strongest association with psychological stress (r = 0.53, *p* < 0.01). Life satisfaction was negatively correlated with all parenting stress subdomains (r = −0.28 to −0.40, *p* < 0.01), indicating that higher stress levels were associated with lower life satisfaction. The correlations, means, and standard deviations among parenting stress subdomains and life satisfaction are presented in Table 4.

### 3.2. Differences by Gender

Variance-robust Welch-type tests revealed significant gender differences in social stress, F(1, 210.139) = 17.609, *p* < 0.001, η^2^ = 0.055, and psychological stress, F(1, 186.714) = 10.185, *p* = 0.002, η^2^ = 0.036. Female parents reported higher levels in both domains. No significant gender differences were observed in economic stress, physical stress, or life satisfaction (all *p* > 0.05).

Supplementary ANCOVA analyses confirmed that gender differences in social stress (F = 22.991, *p* < 0.001) and psychological stress (F = 13.176, *p* < 0.001) remained significant after controlling for household income, education level, disability severity, and duration of participation, supporting the robustness of these effects.

The effect sizes for gender differences were small (social stress: η^2^ = 0.055; psychological stress: η^2^ = 0.036), indicating that these statistically significant differences accounted for a modest proportion of variance. These findings should therefore be interpreted as preliminary evidence of gender-related subgroup differences among APA-receiving families.

### 3.3. Differences by Age

Significant age differences were found in economic stress, F(2, 88.289) = 4.449, *p* = 0.014, η^2^ = 0.031, and physical stress, F(2, 87.291) = 22.961, *p* < 0.001, η^2^ = 0.152.

Games–Howell post hoc comparisons indicated that parents in their 30s reported higher economic stress than those in their 40s (*p* = 0.040) and 50s (*p* = 0.011), whereas no significant difference was observed between the 40s and 50s groups (*p* = 0.553).

For physical stress, parents in their 50s reported significantly higher levels than those in their 40s (*p* < 0.001) and 30s (*p* < 0.001), and parents in their 40s reported higher levels than those in their 30s (*p* = 0.018). No significant age differences were found for social stress, psychological stress, or life satisfaction (all *p* > 0.05).

Supplementary ANCOVA analyses indicated that age differences in physical stress remained significant after controlling for household income, education level, disability severity, and duration of participation (F = 8.282, *p* < 0.001), confirming the robustness of this effect. However, age differences in economic stress were no longer significant after covariate adjustment (F = 2.236, *p* = 0.109), suggesting that the previously observed differences may have been partially attributable to socioeconomic factors, particularly household income, rather than age per se.

Physical stress differences by age demonstrated a medium-to-large effect (η^2^ = 0.152), whereas the small effect size for economic stress (η^2^ = 0.031) and its attenuation after covariate adjustment suggest that age-related differences in economic stress should be interpreted cautiously.

### 3.4. Differences by Education Level

In the primary analyses, educational level was associated with physical stress, F(2, 32.228) = 19.239, *p* < 0.001, η^2^ = 0.118; social stress, F(2, 31.672) = 3.537, raw *p* = 0.041, FDR-adjusted *p* = 0.055, η^2^ = 0.024; psychological stress, F(2, 32.772) = 3.626, raw *p* = 0.038, FDR-adjusted *p* = 0.076, η^2^ = 0.026; and life satisfaction, F(2, 32.023) = 15.510, *p* < 0.001, η^2^ = 0.110. After FDR adjustment, education-related differences remained significant for physical stress and life satisfaction, whereas social and psychological stress differences were attenuated and interpreted as exploratory.

Post hoc analyses showed that parents with a high school education or lower reported higher physical stress than those with university education (*p* < 0.001) and graduate education (*p* < 0.001), whereas the university and graduate groups did not differ significantly (*p* = 0.088). Life satisfaction differed significantly across all three education levels (all *p* < 0.05), with higher education associated with higher life satisfaction. For social stress, the university group reported higher levels than the graduate group (*p* = 0.042). For psychological stress, the high school group reported higher levels than the graduate group (*p* = 0.027). However, because the omnibus findings for social and psychological stress were attenuated after FDR adjustment, these post hoc differences were interpreted as exploratory.

Supplementary ANCOVA analyses indicated that education-level differences in physical stress (F = 14.778, *p* < 0.001) and life satisfaction (F = 19.342, *p* < 0.001) remained significant after controlling for household income, disability severity, and duration of participation, confirming the robustness of these effects. However, education-level differences in social stress (F = 2.711, *p* = 0.068) and psychological stress (F = 3.008, *p* = 0.051) were no longer statistically significant after covariate adjustment, suggesting that these differences may have been partially explained by household income and disability severity.

Among the education-related findings, physical stress (η^2^ = 0.118) and life satisfaction (η^2^ = 0.110) demonstrated medium effect sizes. The small effect sizes for social stress (η^2^ = 0.024) and psychological stress (η^2^ = 0.026), combined with attenuation after FDR adjustment, attenuation after covariate adjustment, and the small graduate-school subgroup (n = 14), indicate that these findings require cautious interpretation.

### 3.5. Differences by Monthly Household Income

Monthly household income showed significant differences in economic stress, F(3, 57.017) = 44.971, *p* < 0.001, η^2^ = 0.31; social stress, F(3, 55.966) = 16.655, *p* < 0.001, η^2^ = 0.15; and life satisfaction, F(3, 53.891) = 10.233, *p* < 0.001, η^2^ = 0.14. No significant differences were found for physical or psychological stress (all *p* > 0.05).

Games–Howell comparisons indicated that economic stress was significantly higher in the $1500–$2300 and $2300–$3100 groups than in the $3100–$3900 and $3900 or above groups (all *p* < 0.001). The $3100–$3900 group also reported higher economic stress than the $3900 or above group (*p* < 0.001), whereas no significant difference was found between the two lowest-income groups (*p* = 0.286).

For social stress, the $2300–$3100 group reported higher levels than the $3100–$3900 (*p* = 0.011) and $3900 or above groups (*p* < 0.001). The $3100–$3900 group also reported higher levels than the $3900 or above group (*p* < 0.001).

For life satisfaction, the $1500–$2300 group reported lower levels than the $2300–$3100 (*p* = 0.044), $3100–$3900 (*p* < 0.001), and $3900 or above groups (*p* < 0.001). Differences among the higher-income groups were not statistically significant.

Supplementary ANCOVA analyses confirmed that income group differences in economic stress (F = 46.840, *p* < 0.001), social stress (F = 20.203, *p* < 0.001), and life satisfaction (F = 18.816, *p* < 0.001) remained significant after controlling for education level, disability severity, and duration of participation, indicating that income-related subgroup differences remained robust after adjustment for education level, disability severity, and duration of participation.

The effect sizes for income-related differences were large for economic stress (η^2^ = 0.310) and medium-to-large for social stress (η^2^ = 0.150) and life satisfaction (η^2^ = 0.140). These findings indicate robust income-related subgroup differences, but they should not be interpreted as causal effects of income in the absence of longitudinal data.

### 3.6. Differences by Child Disability Type

No significant differences across Autism spectrum disorder, Intellectual disability, Physical disability, and Emotional/behavioral disorder were observed in economic, physical, social, or psychological stress (all *p* > 0.05). In the primary analysis, life satisfaction differed by disability type, F(3, 33.655) = 3.433, raw *p* = 0.028, η^2^ = 0.030; however, this association was attenuated after FDR adjustment (FDR-adjusted *p* = 0.056). Given the small sample sizes in the physical disability (n = 12) and emotional/behavioral disorder (n = 15) groups, this finding was interpreted as exploratory.

Post hoc comparisons indicated that parents of children with autism spectrum disorder reported higher life satisfaction than those with intellectual disability (*p* = 0.040) and physical disability (*p* = 0.036). No other pairwise differences were significant. Because the omnibus association was attenuated after FDR adjustment, these pairwise differences should also be interpreted as exploratory.

Disability type was excluded from supplementary ANCOVA analyses to ensure model stability because of the small sample sizes in the physical disability (n = 12) and emotional/behavioral disorder (n = 15) groups.

### 3.7. Differences by Disability Severity

Disability severity (Mild, Moderate, Severe) was significantly associated with physical stress, F(2, 89.738) = 9.837, *p* < 0.001, η^2^ = 0.09; social stress, F(2, 92.454) = 11.170, *p* < 0.001, η^2^ = 0.10; psychological stress, F(2, 97.265) = 22.588, *p* < 0.001, η^2^ = 0.18; and life satisfaction, F(2, 87.391) = 15.110, *p* < 0.001, η^2^ = 0.13. Economic stress was not significant (*p* = 0.172).

Post hoc analyses indicated that the Severe group reported higher physical and social stress than the Moderate and Mild groups (all *p* < 0.01), whereas the Moderate and Mild groups did not differ significantly. For psychological stress, the Severe group reported higher levels than both the Moderate and Mild groups (both *p* < 0.001), and the Moderate group reported higher levels than the Mild group (*p* = 0.007). For life satisfaction, the Severe group reported lower levels than both the Moderate and Mild groups (both *p* < 0.001).

Supplementary ANCOVA analyses confirmed that disability severity differences in physical stress (F = 5.784, *p* = 0.003), social stress (F = 14.713, *p* < 0.001), psychological stress (F = 19.469, *p* < 0.001), and life satisfaction (F = 23.724, *p* < 0.001) remained significant after controlling for household income, education level, and duration of participation, supporting the robustness of these effects. Notably, disability severity also became significantly associated with economic stress after covariate adjustment (F = 6.463, *p* = 0.002). This adjustment-sensitive pattern suggests that severity-related differences in economic stress should be interpreted cautiously and should not be treated as evidence of a formal suppression mechanism.

Effect sizes for disability severity ranged from small-to-medium to medium-to-large across outcomes (psychological stress: η^2^ = 0.180; life satisfaction: η^2^ = 0.130; social stress: η^2^ = 0.100; physical stress: η^2^ = 0.090). These findings suggest that severity-related subgroup differences are important, although the severe subgroup was relatively small (n = 33) and the results should be interpreted cautiously.

### 3.8. Differences by Service Utilization Characteristics

#### 3.8.1. Duration of Participation

Significant differences were observed in economic stress, F(4, 33.529) = 4.076, *p* = 0.008, η^2^ = 0.04; physical stress, F(4, 33.079) = 8.044, *p* < 0.001, η^2^ = 0.12; and social stress, F(4, 33.412) = 7.200, *p* < 0.001, η^2^ = 0.08. No significant differences were found for psychological stress or life satisfaction (all *p* > 0.05).

Post hoc analyses showed that parents with participation durations of 3 years or longer reported higher physical stress than those in the 1–2 years and 2–3 years groups (both *p* < 0.05). No consistent pairwise differences were observed for economic stress.

Supplementary ANCOVA analyses indicated that participation duration differences in physical stress (F = 6.946, *p* < 0.001) and social stress (F = 3.697, *p* = 0.006) remained significant after controlling for household income, education level, and disability severity, supporting the robustness of these effects. However, duration differences in economic stress were no longer significant after covariate adjustment (F = 0.867, *p* = 0.484), suggesting that these differences may have been attributable to income-related factors rather than participation duration per se. Notably, participation duration became significantly associated with psychological stress after covariate adjustment (F = 2.900, *p* = 0.022). This adjustment-sensitive result should be interpreted cautiously because it emerged only after covariate adjustment.

#### 3.8.2. Monthly Treatment Cost

Monthly treatment cost was significantly associated with economic stress, F(4, 105.305) = 6.494, *p* < 0.001, η^2^ = 0.10, and social stress, F(4, 106.929) = 6.464, *p* < 0.001, η^2^ = 0.09. No significant differences were observed for physical stress, psychological stress, or life satisfaction.

Games–Howell comparisons indicated that the $80–$115 group reported higher economic stress than the $155–$195 (*p* = 0.010) and $195 or above groups (*p* < 0.001). For social stress, the $80–$115 group reported higher levels than the $115–$155 (*p* = 0.017), $155–$195 (*p* = 0.001), and $195 or above groups (*p* < 0.001).

Supplementary ANCOVA analyses indicated that treatment cost differences in social stress remained significant after controlling for household income, education level, disability severity, and duration of participation (F = 4.247, *p* = 0.002), confirming the robustness of this effect. However, treatment cost differences in economic stress were no longer significant after covariate adjustment (F = 2.008, *p* = 0.093), suggesting that the previously observed differences may have been largely attributable to household income rather than treatment cost per se. Notably, treatment cost became significantly associated with physical stress after covariate adjustment (F = 3.250, *p* = 0.012). This adjustment-sensitive result should be interpreted cautiously because it was not significant in the primary analysis.

Effect sizes for service-utilization differences were generally small to medium (physical stress: η^2^ = 0.120; social stress: η^2^ = 0.080–0.090; economic stress: η^2^ = 0.040–0.100). These findings suggest modest subgroup differences associated with service duration and out-of-pocket cost; however, causal or cumulative interpretations cannot be made from the present cross-sectional data. A summary of the Welch’s ANOVA results, FDR-adjusted p-values, supplementary ANCOVA results, and effect-size estimates is presented in Table 5.

After Benjamini-Hochberg FDR adjustment within each outcome family, the strongest subgroup differences generally remained statistically significant, but the effect-size estimates and confidence intervals indicated varying degrees of precision. Household income showed the largest and most stable association with economic stress. Gender differences in social and psychological stress, age-related differences in physical stress, education-related differences in physical stress and life satisfaction, and disability severity differences in several outcomes also remained statistically significant after adjustment. In contrast, education-related differences in social and psychological stress, disability-type differences in life satisfaction, duration-related economic stress, and treatment-cost-related economic stress were attenuated or non-robust. Findings that emerged only after covariate adjustment, including severity-related economic stress, duration-related psychological stress, and treatment-cost-related physical stress, were interpreted as adjustment-sensitive rather than as stable subgroup effects.

## 4. Discussion

### 4.1. Multidimensional Stress, Socioeconomic Context, and Disability-Related Caregiving Burden

The present study identified subgroup differences in parenting stress and life satisfaction among parents of children receiving APA-based developmental rehabilitation services. Rather than indicating causal effects of APA participation, the findings suggest that caregiver well-being in this service-using population is shaped by overlapping socioeconomic, disability-related, and service-utilization contexts.

A central finding is that parenting stress was multidimensional and inversely associated with life satisfaction. Economic, physical, social, and psychological stress were interrelated, but each domain appeared to reflect somewhat different aspects of caregiving burden. This pattern is consistent with the transactional view that stress emerges when caregiving demands exceed available resources; however, because appraisal, coping strategies, caregiving hours, and informal support were not directly measured, the model should be understood as an interpretive framework rather than a tested causal mechanism.

Socioeconomic context appeared particularly important. Lower household income was most strongly associated with economic stress and was also related to social stress and life satisfaction. These patterns may reflect cumulative caregiving burden in which financial instability constrains access to paid support, flexible services, transportation options, leisure opportunities, and social participation. Thus, economic vulnerability may operate not only as a direct financial strain but also as a condition that narrows the resources available to families managing long-term disability-related care.

Disability severity was also associated with several stress domains and life satisfaction. Higher psychological and social stress among parents of children with more severe disabilities may reflect long-term caregiving burden, emotional exhaustion, behavioral-management demands, and uncertainty regarding the child’s future support needs. At the same time, severity-related findings should be interpreted cautiously because the severe subgroup was relatively small and because child-level variables such as functional support needs, comorbidities, school level, and behavioral difficulties were not directly measured.

Gender-related differences in social and psychological stress remained after adjustment, but these findings should not be read as evidence of a specific mechanism of maternal burden. Although measurement invariance results supported cautious gender comparisons, the CFA and invariance models were estimated in the same sample used for subgroup inference. In addition, the study did not measure primary caregiver status, caregiving hours, employment, marital status, or informal support. Therefore, gender differences are best interpreted as subgroup patterns requiring replication and further explanation.

Service-utilization findings require particular caution. Longer participation duration and out-of-pocket treatment cost were associated with selected stress domains, but the study did not include comparison groups receiving other rehabilitation services or no services, nor did it directly assess APA-specific service characteristics. Duration and cost may be confounded by disability severity, family resources, service availability, travel demands, and unmeasured program-level factors. Accordingly, the results should not be interpreted as showing that APA services themselves caused or uniquely produced the observed stress patterns.

### 4.2. Potential Implications for Family-Centered Support in Developmental Rehabilitation Service Contexts

The findings suggest that family-centered support within developmental rehabilitation service contexts should consider caregiver well-being alongside child-focused outcomes. Rather than defining definitive intervention targets, the results identify parent subgroups who may warrant further assessment, including mothers reporting higher social or psychological stress, families with lower household income, and parents of children with more severe disabilities.

Support strategies should be framed broadly rather than as APA-specific prescriptions. Caregiver screening, referral information, peer support, and stress-management education may be useful components of family-centered service systems, but future studies must determine whether these approaches improve caregiver outcomes and whether their effects differ across service modalities.

Financial vulnerability and functional support needs also warrant attention in future service-level research. Studies that include transportation costs, session frequency, parental involvement, travel time, instructor characteristics, and comparison groups from other rehabilitation services are needed before conclusions can be drawn about which service features are associated with caregiver stress or life satisfaction.

## 5. Limitations and Future Research

First, the cross-sectional design precludes causal and temporal interpretations. The study identified subgroup differences and associations, but it cannot determine whether APA participation, service duration, treatment cost, or disability-related characteristics caused changes in parenting stress or life satisfaction.

Second, the use of convenience sampling from 16 child development centers offering APA-based developmental rehabilitation services limits generalizability. Although the response rate and valid response rate were reported, the sample may not represent parents who do not use APA services, who use other rehabilitation modalities, who discontinued services, or who face barriers to service access.

Third, all data were based on parent self-report, which may introduce common method bias, recall bias, and social desirability bias. Future studies should combine parent reports with qualitative interviews, provider reports, administrative service records, and objective program-level indicators.

Fourth, several important parent-level and child-level covariates were not measured, including employment status, marital status, single-parent status, primary caregiver status, caregiving hours, number of children, parent physical health, parent mental health history, informal support, child age, child sex, school level, comorbidities, and functional support needs. These omitted variables may confound some of the observed associations.

Fifth, although the adapted Parenting Stress Index demonstrated acceptable internal consistency, a four-factor structure in CFA, and gender-based measurement invariance in the current sample, it has not undergone full construct validation in an independent sample. The psychological stress subscale included only three items and showed acceptable but comparatively lower reliability (α = 0.77). Therefore, further validation in independent and more diverse samples is warranted. Because the CFA and invariance analyses were conducted in the same sample used for subgroup comparisons, these results should be regarded as supportive but not confirmatory evidence of measurement validity.

Sixth, the study involved numerous subgroup comparisons. Although Benjamini–Hochberg FDR adjustment was applied to reduce the risk of Type I error inflation, the findings should remain exploratory until replicated in confirmatory studies. Some subgroup results were based on small cell sizes, including graduate education, physical disability, emotional/behavioral disorder, and severe disability groups, which limits inferential stability.

Seventh, service-level characteristics were not measured. Variables such as session frequency, session duration, travel time, class size, instructor qualifications, instructional methods, and required parental involvement may influence both service participation and caregiver well-being. Future studies should incorporate objective program-level measures and comparison groups from other rehabilitation modalities.

Finally, the findings should be interpreted within the institutional context of South Korea’s Developmental Rehabilitation Service system. Generalizability to other countries or service systems may be limited.

## 6. Conclusions

This exploratory cross-sectional study examined subgroup differences in parenting stress and life satisfaction among parents of children with disabilities receiving adapted physical activity services. Parenting stress domains were positively interrelated and negatively associated with life satisfaction, and selected differences were observed according to gender, household income, disability severity, and service-utilization characteristics. However, because the study was based on self-reported data, convenience sampling, and an adapted measure that requires further validation, the findings should be interpreted as preliminary associative evidence. The results do not establish that APA services cause, reshape, or accumulate caregiver burden over time. Instead, they suggest that APA service settings may be useful contexts for future research on caregiver well-being, particularly studies using longitudinal, comparative, and service-level designs.

## Figures and Tables

**Table 1:** Participant characteristics (N = 295).

Characteristic	Category	n	%
Gender of parent	Male	101	34.2
	Female	194	65.8
Age	30–39 years	35	11.9
	40–49 years	174	59.0
	50 years and above	86	29.1
Education level	High school or less	45	15.3
	University	236	80.0
	Graduate school	14	4.7
Monthly household income	$1500~$2300	14	4.7
	$2300~$3100	66	22.4
	$3100~$3900	140	47.5
	$3900 or above	75	25.4
Child’s disability type	Autism spectrum disorder	159	53.9
	Intellectual disability	109	36.9
	Physical disability	12	4.1
	Emotional/behavioral disorder	15	5.1
Severity of disability	Mild	113	38.3
	Moderate	149	50.5
	Severe	33	11.2

**Table 2:** Service Utilization Characteristics of Participants.

Characteristic	Category	n	%
Duration of Participation	Less than 6 months	6	2.0
	6–12 months	29	9.8
	1–2 years	66	22.4
	2–3 years	89	30.2
	3 years or more	105	35.6
Monthly Treatment Cost	Less than $80	23	7.8
	$80~$115	69	23.4
	$115~$155	62	21.0
	$155~$195	70	23.7
	$195 or above	71	24.1

**Table 3:** Factor Loadings and Reliability Coefficients for Parenting Stress and Life Satisfaction.

Factors/Items	λ	α	ω
**Economic Stress**		0.85	0.89
I feel burdened due to the financial costs associated with caring for my child with a disability.	0.87		
Because of my child’s disability, I have had to reduce my personal financial spending.	0.85		
I would like to reduce the expenses incurred for my child with a disability.	0.81		
Due to expenses related to my child’s disability, I am unable to adequately prepare for my future financial security.	0.71		
**Psychological Stress**		0.77	0.79
I want to do well for my child, but I often feel distressed when I feel I cannot meet their needs.	0.79		
When I think about my child’s difficulties in leading a typical life, I feel sorrowful.	0.75		
I sometimes feel guilty about my child’s disability.	0.70		
**Physical Stress**		0.83	0.90
When I think about my child with a disability, I experience physical symptoms such as indigestion, nausea, or headaches.	0.91		
My own health condition makes it difficult for me to care for my child.	0.85		
When I think about my child with a disability, I feel tightness or discomfort in my chest.	0.80		
When I think about my child with a disability, I feel my heart beating rapidly.	0.75		
**Social Stress**		0.80	0.82
Caring for my child with a disability often requires me to give up things I would normally enjoy.	0.81		
Because of caring responsibilities, I find it difficult to maintain social relationships.	0.77		
The time required to care for my child interferes with my social life.	0.74		
**Life Satisfaction**		0.87	0.92
I often feel joy and happiness in my life.	0.88		
I believe that my life is valuable and meaningful.	0.86		
I believe that my life is moving in a positive and hopeful direction.	0.80		
I feel a sense of fulfillment and achievement in my current life.	0.80		
Overall, I am satisfied with my life.	0.79		

Note. Bold text indicates factor/domain labels. λ = standardized factor loading; α = Cronbach’s alpha; ω = McDonald’s omega.

**Table 4:** Correlations, means, and SD among parenting stress and life satisfaction.

Scales		1	2	3	4	5
1.	Economic Stress	1				
2.	Physical Stress	0.19 **	1			
3.	Social Stress	0.46 **	0.20 **	1		
4.	Psychological Stress	0.33 **	0.37 **	0.53 **	1	
5.	Life Satisfaction	−0.40 **	−0.28 **	−0.30 **	−0.39 **	1
	*M*	2.84	2.56	3.23	2.56	3.78
	*SD*	0.89	0.85	0.85	0.85	0.63

Note. Correlations are presented for the full sample. Gender-stratified correlations and formal tests of gender differences in correlation coefficients were not conducted because the primary focus of the study was subgroup mean differences rather than moderation of associations. ** *p* < 0.01.

**Table 5:** Summary of Welch’s ANOVA, FDR Adjustment, and Supplementary ANCOVA for Parenting Stress and Life Satisfaction.

Factor	Variable	F (df1, df2)	Raw *p*	FDR -Adjusted *p*	η^2^	ANCOVA F	ANCOVA *p*	Robust	η^2^ 95% CI	ω^2^
Gender	Social Stress	F(1, 210.14) = 17.609	<0.001	<0.001	0.055	22.991	<0.001	✓	0.020–0.095	0.051
Gender	Psychological Stress	F(1, 186.71) = 10.185	0.002	0.008	0.036	13.176	<0.001	✓	0.006–0.075	0.032
Age	Economic Stress	F(2, 88.29) = 4.449	0.014	0.028	0.031	2.236	0.109	✗	0.002–0.075	0.024
Age	Physical Stress	F(2, 87.29) = 22.961	<0.001	<0.001	0.152	8.282	<0.001	✓	0.085–0.220	0.145
Education	Physical Stress	F(2, 32.23) = 19.239	<0.001	<0.001	0.118	14.778	<0.001	✓	0.050–0.200	0.111
Education	Social Stress	F(2, 31.67) = 3.537	0.041	0.055	0.024	2.711	0.068	✗	0.000–0.065	0.017
Education	Psychological Stress	F(2, 32.77) = 3.626	0.038	0.076	0.026	3.008	0.051	✗	0.000–0.070	0.019
Education	Life Satisfaction	F(2, 32.02) = 15.510	<0.001	<0.001	0.110	19.342	<0.001	✓	0.045–0.190	0.103
Income	Economic Stress	F(3, 57.02) = 44.971	<0.001	<0.001	0.310	46.840	<0.001	✓	0.220–0.390	0.299
Income	Social Stress	F(3, 55.97) = 16.655	<0.001	<0.001	0.150	20.203	<0.001	✓	0.075–0.230	0.139
Income	Life Satisfaction	F(3, 53.89) = 10.233	<0.001	<0.001	0.140	18.816	<0.001	✓	0.060–0.220	0.129
Disability Type	Life Satisfaction	F(3, 33.66) = 3.433	0.028	0.056	0.030	—	—	✗ †	0.000–0.090	0.020
Severity	Economic Stress	—	0.172	—	—	6.463	0.002	↑	—	—
Severity	Physical Stress	F(2, 89.74) = 9.837	<0.001	<0.001	0.090	5.784	0.003	✓	0.035–0.155	0.083
Severity	Social Stress	F(2, 92.45) = 11.170	<0.001	<0.001	0.100	14.713	<0.001	✓	0.040–0.170	0.093
Severity	Psychological Stress	F(2, 97.27) = 22.588	<0.001	<0.001	0.180	19.469	<0.001	✓	0.105–0.255	0.173
Severity	Life Satisfaction	F(2, 87.39) = 15.110	<0.001	<0.001	0.130	23.724	<0.001	✓	0.060–0.205	0.123
Duration	Economic Stress	F(4, 33.53) = 4.076	0.008	0.021	0.040	0.867	0.484	✗	0.000–0.095	0.026
Duration	Physical Stress	F(4, 33.08) = 8.044	<0.001	<0.001	0.120	6.946	<0.001	✓	0.045–0.205	0.106
Duration	Social Stress	F(4, 33.41) = 7.200	<0.001	<0.001	0.080	3.697	0.006	✓	0.020–0.155	0.066
Duration	Psychological Stress	—	>0.05	—	—	2.900	0.022	↑	—	—
Treatment Cost	Economic Stress	F(4, 105.31) = 6.494	<0.001	<0.001	0.100	2.008	0.093	✗	0.035–0.180	0.086
Treatment Cost	Physical Stress	—	>0.05	—	—	3.250	0.012	↑	—	—
Treatment Cost	Social Stress	F(4, 106.93) = 6.464	<0.001	<0.001	0.090	4.247	0.002	✓	0.030–0.170	0.076

Note. Raw *p*-values refer to Welch’s ANOVA results. FDR-adjusted *p*-values were calculated using the Benjamini-Hochberg procedure within each outcome family to account for multiple subgroup comparisons. η^2^ values are reported with bootstrapped 95% confidence intervals, and ω^2^ is reported as a sensitivity estimate for omnibus group differences. Effect-size estimates under Welch-type tests should be interpreted cautiously. ANCOVA covariates varied by factor, as described in the Methods section. ✓ = robust finding that remained statistically significant after both FDR adjustment and covariate adjustment; ✗ = finding that was no longer statistically significant after FDR adjustment and/or covariate adjustment; ↑ = finding that was not significant in the primary analysis but became significant after covariate adjustment and was therefore interpreted as adjustment-sensitive; — = not applicable or not significant in the primary analysis. † Disability type was excluded from ANCOVA due to small subgroup sizes (n = 12–15). Complete raw and FDR-adjusted *p*-values for all subgroup comparisons are provided in Supplementary Table S2.

## Data Availability

The data presented in this study are not publicly available due to privacy and ethical restrictions related to sensitive information from participants. However, the data may be made available from the corresponding author upon reasonable request and with permission from the Institutional Review Board.

## Supplementary Materials

The following supporting information can be downloaded at: https://www.mdpi.com/article/10.3390/healthcare14111434/s1, Table S1: Confirmatory Factor Analysis and Gender-Based Measurement Invariance of the Adapted Parenting Stress Scale; Table S2: Complete Raw and FDR-Adjusted *p*-Values for Subgroup Comparisons within Each Outcome Family; Table S3: Subgroup Descriptive Statistics for Parenting Stress and Life Satisfaction; STROBE Checklist: STROBE Statement—Checklist of Items for Cross-Sectional Studies.
