# Supplementary material for: Subgroup Differences in Parenting Stress and Life Satisfaction Among Parents of Children with Disabilities Receiving Adapted Physical Activity Services
Source: Healthcare (Basel). 2026 May 22;14(11):1434. doi: 10.3390/healthcare14111434 (PMC13257220; doi:10.3390/healthcare14111434)
Supplement: Supplementary file 1 [file healthcare-14-01434-s001.zip › healthcare-4283398-supplementary.pdf]

Supplementary Table S1. Confirmatory Factor Analysis and Gender-Based Measurement Invariance of the Adapted Parenting Stress Scale

Model	χ^2	df	χ^2/df	CFI	TLI	RMSEA	SRMR	ΔCFI	ΔRMSEA
Four-factor CFA model	142.36	71	2.01	.933	.929	.058	.046	—	—
Configural invariance	223.48	142	1.57	.938	.922	.062	.053	—	—
Metric invariance	236.91	152	1.56	.936	.925	.061	.058	-.002	-.001
Scalar invariance	253.27	162	1.56	.932	.924	.062	.060	-.004	.001

Note. The four-factor CFA model was estimated using the full sample. Gender-based measurement invariance was tested across male and female parents. ΔCFI and ΔRMSEA were calculated relative to the preceding, less constrained model. Changes in CFI $\leq$ .010 and RMSEA $\leq$ .015 were interpreted as supporting measurement invariance.

Supplementary Table S2. Complete Raw and FDR-Adjusted p-Values for Subgroup Comparisons within Each Outcome Family

Outcome family	Factor	Raw *p*	FDR-adjusted *p*	Interpretation
Economic stress	Gender	.417	.477	Not significant
Economic stress	Age	.014	.028	Retained after FDR; non-robust after ANCOVA
Economic stress	Education	.098	.131	Not significant
Economic stress	Household income	<.001	<.001	Retained after FDR
Economic stress	Disability type	.742	.742	Not significant
Economic stress	Disability severity	.172	.229	Not significant in primary analysis; significant only after ANCOVA
Economic stress	Duration of participation	.008	.021	Retained after FDR; non-robust after ANCOVA
Economic stress	Monthly treatment cost	<.001	<.001	Retained after FDR; non-robust after ANCOVA
Physical stress	Gender	.326	.435	Not significant
Physical stress	Age	<.001	<.001	Retained after FDR and ANCOVA
Physical stress	Education	<.001	<.001	Retained after FDR and ANCOVA
Physical stress	Household income	.412	.471	Not significant
Physical stress	Disability type	.586	.586	Not significant
Physical stress	Disability severity	<.001	<.001	Retained after FDR and ANCOVA
Physical stress	Duration of participation	<.001	<.001	Retained after FDR and ANCOVA
Physical stress	Monthly treatment cost	.117	.187	Not significant in primary analysis; significant only after ANCOVA
Social stress	Gender	<.001	<.001	Retained after FDR and ANCOVA
Social stress	Age	.286	.327	Not significant
Social stress	Education	.041	.055	Attenuated after FDR; exploratory
Social stress	Household income	<.001	<.001	Retained after FDR and ANCOVA
Social stress	Disability type	.518	.518	Not significant
Social stress	Disability severity	<.001	<.001	Retained after FDR and ANCOVA
Social stress	Duration of participation	<.001	<.001	Retained after FDR and ANCOVA
Social stress	Monthly treatment cost	<.001	<.001	Retained after FDR and ANCOVA
Psychological stress	Gender	.002	.008	Retained after FDR and ANCOVA
Psychological stress	Age	.244	.390	Not significant
Psychological stress	Education	.038	.076	Attenuated after FDR; exploratory
Psychological stress	Household income	.468	.535	Not significant
Psychological stress	Disability type	.692	.692	Not significant
Psychological stress	Disability severity	<.001	<.001	Retained after FDR and ANCOVA
Psychological stress	Duration of participation	.137	.274	Not significant in primary analysis; significant only after ANCOVA
Psychological stress	Monthly treatment cost	.319	.425	Not significant
Life satisfaction	Gender	.648	.648	Not significant
Life satisfaction	Age	.214	.342	Not significant
Life satisfaction	Education	<.001	<.001	Retained after FDR and ANCOVA
Life satisfaction	Household income	<.001	<.001	Retained after FDR and ANCOVA
Life satisfaction	Disability type	.028	.056	Attenuated after FDR; exploratory
Life satisfaction	Disability severity	<.001	<.001	Retained after FDR and ANCOVA
Life satisfaction	Duration of participation	.327	.374	Not significant
Life satisfaction	Monthly treatment cost	.593	.648	Not significant

Note. Raw *p*-values refer to the primary subgroup comparison analyses. FDR-adjusted *p*-values were calculated within each outcome family using the Benjamini–Hochberg procedure. Interpretations indicate whether each subgroup difference remained statistically significant after FDR adjustment and, where applicable, supplementary ANCOVA. Results that were significant only after covariate adjustment were interpreted as adjustment-sensitive. ANCOVA = analysis of covariance; FDR = false discovery rate.

Supplementary Table S3. Subgroup Descriptive Statistics for Parenting Stress and Life Satisfaction

Grouping factor	Subgroup	Outcome	n	M	SD	SE	95% CI lower	95% CI upper
Gender	Male	Economic stress	101	2.91	0.89	0.09	2.74	3.08
Gender	Female	Economic stress	194	2.80	0.87	0.06	2.68	2.92
Gender	Male	Physical stress	101	2.50	0.85	0.08	2.33	2.67
Gender	Female	Physical stress	194	2.59	0.83	0.06	2.47	2.71
Gender	Male	Social stress	101	2.95	0.83	0.08	2.79	3.11
Gender	Female	Social stress	194	3.37	0.81	0.06	3.26	3.48
Gender	Male	Psychological stress	101	2.34	0.83	0.08	2.18	2.50
Gender	Female	Psychological stress	194	2.68	0.81	0.06	2.57	2.79
Gender	Male	Life satisfaction	101	3.82	0.63	0.06	3.70	3.94
Gender	Female	Life satisfaction	194	3.76	0.62	0.04	3.67	3.85
Age	30-39 years	Economic stress	35	3.26	0.88	0.15	2.97	3.55
Age	40-49 years	Economic stress	174	2.81	0.86	0.07	2.68	2.94
Age	50 years and above	Economic stress	86	2.74	0.88	0.09	2.55	2.93
Age	30-39 years	Physical stress	35	2.01	0.78	0.13	1.75	2.27
Age	40-49 years	Physical stress	174	2.44	0.76	0.06	2.33	2.55
Age	50 years and above	Physical stress	86	3.03	0.78	0.08	2.87	3.19
Age	30-39 years	Social stress	35	3.34	0.85	0.14	3.06	3.62
Age	40-49 years	Social stress	174	3.23	0.83	0.06	3.11	3.35
Age	50 years and above	Social stress	86	3.18	0.85	0.09	3.00	3.36
Age	30-39 years	Psychological stress	35	2.67	0.85	0.14	2.39	2.95
Age	40-49 years	Psychological stress	174	2.56	0.83	0.06	2.44	2.68
Age	50 years and above	Psychological stress	86	2.51	0.85	0.09	2.33	2.69
Age	30-39 years	Life satisfaction	35	3.70	0.63	0.11	3.49	3.91
Age	40-49 years	Life satisfaction	174	3.81	0.62	0.05	3.72	3.90
Age	50 years and above	Life satisfaction	86	3.75	0.63	0.07	3.62	3.88
Education level	High school or less	Economic stress	45	3.05	0.88	0.13	2.79	3.31
Education level	University	Economic stress	236	2.81	0.86	0.06	2.70	2.92
Education level	Graduate school	Economic stress	14	2.62	0.91	0.24	2.14	3.10
Education level	High school or less	Physical stress	45	3.22	0.80	0.12	2.99	3.45
Education level	University	Physical stress	236	2.46	0.78	0.05	2.36	2.56
Education level	Graduate school	Physical stress	14	2.06	0.83	0.22	1.63	2.49
Education level	High school or less	Social stress	45	3.29	0.84	0.13	3.04	3.54
Education level	University	Social stress	236	3.25	0.82	0.05	3.15	3.35
Education level	Graduate school	Social stress	14	2.64	0.87	0.23	2.18	3.10
Education level	High school or less	Psychological stress	45	2.81	0.84	0.13	2.56	3.06
Education level	University	Psychological stress	236	2.54	0.82	0.05	2.44	2.64
Education level	Graduate school	Psychological stress	14	2.13	0.87	0.23	1.67	2.59
Education level	High school or less	Life satisfaction	45	3.38	0.59	0.09	3.21	3.55
Education level	University	Life satisfaction	236	3.82	0.58	0.04	3.75	3.89
Education level	Graduate school	Life satisfaction	14	4.39	0.61	0.16	4.07	4.71
Monthly household income	$1,500-$2,300	Economic stress	14	3.74	0.77	0.21	3.34	4.14
Monthly household income	$2,300-$3,100	Economic stress	66	3.49	0.74	0.09	3.31	3.67
Monthly household income	$3,100-$3,900	Economic stress	140	2.80	0.74	0.06	2.68	2.92
Monthly household income	$3,900 or above	Economic stress	75	2.18	0.74	0.09	2.01	2.35
Monthly household income	$1,500-$2,300	Physical stress	14	2.71	0.88	0.24	2.25	3.17
Monthly household income	$2,300-$3,100	Physical stress	66	2.58	0.85	0.10	2.37	2.79
Monthly household income	$3,100-$3,900	Physical stress	140	2.52	0.85	0.07	2.38	2.66
Monthly household income	$3,900 or above	Physical stress	75	2.58	0.85	0.10	2.39	2.77
Monthly household income	$1,500-$2,300	Social stress	14	3.47	0.81	0.22	3.05	3.89
Monthly household income	$2,300-$3,100	Social stress	66	3.70	0.78	0.10	3.51	3.89
Monthly household income	$3,100-$3,900	Social stress	140	3.23	0.78	0.07	3.10	3.36

Monthly household income	$3,900 or above	Social stress	75	2.76	0.78	0.09	2.58	2.94
Monthly household income	$1,500-$2,300	Psychological stress	14	2.71	0.88	0.24	2.25	3.17
Monthly household income	$2,300-$3,100	Psychological stress	66	2.58	0.85	0.10	2.37	2.79
Monthly household income	$3,100-$3,900	Psychological stress	140	2.52	0.85	0.07	2.38	2.66
Monthly household income	$3,900 or above	Psychological stress	75	2.58	0.85	0.10	2.39	2.77
Monthly household income	$1,500-$2,300	Life satisfaction	14	2.83	0.60	0.16	2.52	3.14
Monthly household income	$2,300-$3,100	Life satisfaction	66	3.67	0.58	0.07	3.53	3.81
Monthly household income	$3,100-$3,900	Life satisfaction	140	3.83	0.58	0.05	3.73	3.93
Monthly household income	$3,900 or above	Life satisfaction	75	3.96	0.58	0.07	3.83	4.09
Child disability type	Autism spectrum disorder	Economic stress	159	2.82	0.87	0.07	2.68	2.96
Child disability type	Intellectual disability	Economic stress	109	2.88	0.89	0.09	2.71	3.05
Child disability type	Physical disability	Economic stress	12	2.94	0.92	0.27	2.42	3.46
Child disability type	Emotional/behavioral disorder	Economic stress	15	2.69	0.92	0.24	2.22	3.16
Child disability type	Autism spectrum disorder	Physical stress	159	2.54	0.83	0.07	2.41	2.67
Child disability type	Intellectual disability	Physical stress	109	2.60	0.85	0.08	2.44	2.76
Child disability type	Physical disability	Physical stress	12	2.66	0.88	0.25	2.16	3.16
Child disability type	Emotional/behavioral disorder	Physical stress	15	2.42	0.88	0.23	1.97	2.87
Child disability type	Autism spectrum disorder	Social stress	159	3.21	0.83	0.07	3.08	3.34
Child disability type	Intellectual disability	Social stress	109	3.27	0.85	0.08	3.11	3.43
Child disability type	Physical disability	Social stress	12	3.33	0.88	0.25	2.83	3.83
Child disability type	Emotional/behavioral disorder	Social stress	15	3.09	0.88	0.23	2.64	3.54
Child disability type	Autism spectrum disorder	Psychological stress	159	2.54	0.83	0.07	2.41	2.67
Child disability type	Intellectual disability	Psychological stress	109	2.60	0.85	0.08	2.44	2.76
Child disability type	Physical disability	Psychological stress	12	2.66	0.88	0.25	2.16	3.16
Child disability type	Emotional/behavioral disorder	Psychological stress	15	2.42	0.88	0.23	1.97	2.87
Child disability type	Autism spectrum disorder	Life satisfaction	159	3.88	0.61	0.05	3.79	3.97
Child disability type	Intellectual disability	Life satisfaction	109	3.67	0.62	0.06	3.55	3.79
Child disability type	Physical disability	Life satisfaction	12	3.53	0.64	0.18	3.17	3.89
Child disability type	Emotional/behavioral disorder	Life satisfaction	15	3.76	0.64	0.17	3.44	4.08
Disability severity	Mild	Economic stress	113	2.79	0.89	0.08	2.63	2.95
Disability severity	Moderate	Economic stress	149	2.84	0.89	0.07	2.70	2.98
Disability severity	Severe	Economic stress	33	3.02	0.89	0.15	2.72	3.32
Disability severity	Mild	Physical stress	113	2.40	0.81	0.08	2.25	2.55
Disability severity	Moderate	Physical stress	149	2.53	0.81	0.07	2.40	2.66
Disability severity	Severe	Physical stress	33	3.26	0.81	0.14	2.98	3.54
Disability severity	Mild	Social stress	113	3.06	0.81	0.08	2.91	3.21
Disability severity	Moderate	Social stress	149	3.20	0.81	0.07	3.07	3.33
Disability severity	Severe	Social stress	33	3.97	0.81	0.14	3.69	4.25
Disability severity	Mild	Psychological stress	113	2.24	0.77	0.07	2.10	2.38
Disability severity	Moderate	Psychological stress	149	2.61	0.77	0.06	2.49	2.73
Disability severity	Severe	Psychological stress	33	3.45	0.77	0.13	3.19	3.71
Disability severity	Mild	Life satisfaction	113	3.92	0.59	0.06	3.81	4.03
Disability severity	Moderate	Life satisfaction	149	3.82	0.59	0.05	3.73	3.91
Disability severity	Severe	Life satisfaction	33	3.15	0.59	0.10	2.95	3.35
Duration of participation	Less than 6 months	Economic stress	6	3.17	0.90	0.37	2.45	3.89
Duration of participation	6-12 months	Economic stress	29	3.02	0.87	0.16	2.70	3.34
Duration of participation	1-2 years	Economic stress	66	2.63	0.87	0.11	2.42	2.84
Duration of participation	2-3 years	Economic stress	89	2.71	0.87	0.09	2.53	2.89
Duration of participation	3 years or more	Economic stress	105	3.02	0.87	0.08	2.85	3.19
Duration of participation	Less than 6 months	Physical stress	6	2.39	0.83	0.34	1.73	3.05
Duration of participation	6-12 months	Physical stress	29	2.50	0.80	0.15	2.21	2.79
Duration of participation	1-2 years	Physical stress	66	2.27	0.80	0.10	2.08	2.46
Duration of participation	2-3 years	Physical stress	89	2.35	0.80	0.08	2.18	2.52
Duration of participation	3 years or more	Physical stress	105	2.95	0.80	0.08	2.80	3.10
Duration of participation	Less than 6 months	Social stress	6	2.75	0.85	0.35	2.07	3.43

Duration of participation	6-12 months	Social stress	29	2.88	0.82	0.15	2.58	3.18
Duration of participation	1-2 years	Social stress	66	3.01	0.82	0.10	2.81	3.21
Duration of participation	2-3 years	Social stress	89	3.20	0.82	0.09	3.03	3.37
Duration of participation	3 years or more	Social stress	105	3.52	0.82	0.08	3.36	3.68
Duration of participation	Less than 6 months	Psychological stress	6	2.28	0.88	0.36	1.58	2.98
Duration of participation	6-12 months	Psychological stress	29	2.39	0.85	0.16	2.08	2.70
Duration of participation	1-2 years	Psychological stress	66	2.50	0.85	0.10	2.29	2.71
Duration of participation	2-3 years	Psychological stress	89	2.61	0.85	0.09	2.43	2.79
Duration of participation	3 years or more	Psychological stress	105	2.61	0.85	0.08	2.45	2.77
Duration of participation	Less than 6 months	Life satisfaction	6	3.91	0.65	0.27	3.39	4.43
Duration of participation	6-12 months	Life satisfaction	29	3.85	0.63	0.12	3.62	4.08
Duration of participation	1-2 years	Life satisfaction	66	3.79	0.63	0.08	3.64	3.94
Duration of participation	2-3 years	Life satisfaction	89	3.79	0.63	0.07	3.66	3.92
Duration of participation	3 years or more	Life satisfaction	105	3.74	0.63	0.06	3.62	3.86
Monthly treatment cost	Less than $80	Economic stress	23	2.90	0.84	0.18	2.56	3.24
Monthly treatment cost	$80-$115	Economic stress	69	3.26	0.84	0.10	3.06	3.46
Monthly treatment cost	$115-$155	Economic stress	62	2.94	0.84	0.11	2.73	3.15
Monthly treatment cost	$155-$195	Economic stress	70	2.66	0.84	0.10	2.46	2.86
Monthly treatment cost	$195 or above	Economic stress	71	2.50	0.84	0.10	2.30	2.70
Monthly treatment cost	Less than $80	Physical stress	23	2.83	0.85	0.18	2.48	3.18
Monthly treatment cost	$80-$115	Physical stress	69	2.57	0.85	0.10	2.37	2.77
Monthly treatment cost	$115-$155	Physical stress	62	2.50	0.85	0.11	2.29	2.71
Monthly treatment cost	$155-$195	Physical stress	70	2.50	0.85	0.10	2.30	2.70
Monthly treatment cost	$195 or above	Physical stress	71	2.57	0.85	0.10	2.37	2.77
Monthly treatment cost	Less than $80	Social stress	23	3.23	0.81	0.17	2.90	3.56
Monthly treatment cost	$80-$115	Social stress	69	3.67	0.81	0.10	3.48	3.86
Monthly treatment cost	$115-$155	Social stress	62	3.20	0.81	0.10	3.00	3.40
Monthly treatment cost	$155-$195	Social stress	70	3.09	0.81	0.10	2.90	3.28
Monthly treatment cost	$195 or above	Social stress	71	2.98	0.81	0.10	2.79	3.17
Monthly treatment cost	Less than $80	Psychological stress	23	2.56	0.85	0.18	2.21	2.91
Monthly treatment cost	$80-$115	Psychological stress	69	2.65	0.85	0.10	2.45	2.85
Monthly treatment cost	$115-$155	Psychological stress	62	2.56	0.85	0.11	2.35	2.77
Monthly treatment cost	$155-$195	Psychological stress	70	2.52	0.85	0.10	2.32	2.72
Monthly treatment cost	$195 or above	Psychological stress	71	2.52	0.85	0.10	2.32	2.72
Monthly treatment cost	Less than $80	Life satisfaction	23	3.78	0.63	0.13	3.52	4.04
Monthly treatment cost	$80-$115	Life satisfaction	69	3.72	0.63	0.08	3.57	3.87
Monthly treatment cost	$115-$155	Life satisfaction	62	3.84	0.63	0.08	3.68	4.00
Monthly treatment cost	$155-$195	Life satisfaction	70	3.78	0.63	0.08	3.63	3.93
Monthly treatment cost	$195 or above	Life satisfaction	71	3.78	0.63	0.07	3.63	3.93

Note. M = mean; SD = standard deviation; SE = standard error; CI = confidence interval. Confidence intervals are presented as 95% confidence intervals.
