# Supplementary material for: Subgroup Differences in Parenting Stress and Life Satisfaction Among Parents of Children with Disabilities Receiving Adapted Physical Activity Services
Source: Healthcare (Basel). 2026 May 22;14(11):1434. doi: 10.3390/healthcare14111434 (PMC13257220; doi:10.3390/healthcare14111434)
Supplement: Supplementary file 1 [file healthcare-14-01434-s001.zip › healthcare-4283398-STROBE Checklist.pdf]

STROBE Statement—Checklist of Items for Cross-Sectional Studies

Manuscript: Subgroup Differences in Parenting Stress and Life Satisfaction among Parents of Children with Disabilities Receiving Adapted Physical Activity Services

Manuscript ID: healthcare-4283398

Section	Item	STROBE recommendation	Location in manuscript	Reported	Author comment
Title and abstract	1	Indicate the study design with a commonly used term in the title or abstract and provide an informative, balanced summary of the study.	Title; Abstract	Yes	The title identifies the population and outcomes, and the Abstract describes the cross-sectional design, sample, analyses, results, and cautious interpretation.
Introduction	2	Explain the scientific background and rationale for the study.	Introduction, paragraphs 1–6	Yes	The Introduction describes parenting stress, life satisfaction, APA services within the DRS system, and the rationale for examining subgroup differences.
Introduction	3	State specific objectives, including any prespecified hypotheses or research questions.	Introduction, final paragraphs	Yes	The objectives and two research questions are stated explicitly.
Methods	4	Present key elements of the study design early in the paper.	Section 2.1 Participants	Yes	The study is described as a cross-sectional survey design.
Methods	5	Describe the setting, locations, and relevant dates, including recruitment and data collection periods.	Section 2.1 Participants	Yes	The manuscript reports recruitment from 16 child development centers in Busan, Ulsan, Gyeongnam, and Jeju, with data collection beginning in September 2024 over two months.
Methods	6(a)	Give eligibility criteria and the sources and methods of participant selection.	Section 2.1 Participants	Yes	Eligibility criteria, exclusion criteria, convenience sampling, center selection, and participant recruitment procedures are described.
Methods	7	Clearly define outcomes, exposures, predictors, potential confounders, and effect modifiers where applicable.	Sections 2.2–2.4	Yes	Parenting stress subdomains and life satisfaction are defined as outcomes; sociodemographic, disability-related, and service-utilization grouping variables and selected covariates are described.
Methods	8	For each variable of interest, describe sources of data and methods of assessment or measurement.	Sections 2.2 and 2.3	Yes	Service-utilization measures, Parenting Stress Index adaptation, life satisfaction scale, response format, reliability, CFA, and measurement invariance procedures are reported.
Methods	9	Describe efforts to address potential sources of bias.	Sections 2.1, 2.3, 2.4, 5	Yes	The manuscript reports exclusion criteria, listwise deletion, measurement checks, FDR adjustment, supplementary ANCOVA, and limitations related to sampling, self-report, and omitted covariates.
Methods	10	Explain how the study size was arrived at.	Section 2.1 Participants	Partial	The manuscript reports the number approached, number responding, exclusions, final analytic sample, response rate, and valid response rate. A formal a priori sample-size calculation was not reported.
Methods	11	Explain how quantitative variables were handled in the analyses and describe any grouping decisions.	Sections 2.2 and 2.4	Yes	The manuscript describes categorization of duration of participation and treatment cost, including data-driven and policy-relevant bases for cut points.
Methods	12(a)	Describe all statistical methods, including those used to control for confounding.	Section 2.4 Data Analysis	Yes	Welch-type tests, one-way ANOVA, Games–Howell post hoc tests, Pearson correlations, FDR adjustment, effect sizes, and supplementary ANCOVA are described.

Methods	12(b)	Describe methods used to examine subgroups and interactions.	Section 2.4 Data Analysis	Yes	The analytic focus was subgroup comparisons; measurement invariance testing was conducted for gender-related interpretation.
Methods	12(c)	Explain how missing data were addressed.	Section 2.1 Participants	Yes	Missing data were handled using listwise deletion, and only complete cases were retained.
Methods	12(d)	If applicable, describe analytical methods taking account of sampling strategy.	Sections 2.1, 2.4, 5	Partial	Convenience sampling is reported and discussed as a limitation. No complex survey sampling weights or clustered sampling adjustments were applied.
Methods	12(e)	Describe any sensitivity analyses.	Sections 2.3 and 2.4; Table 5; Supplementary Tables S1–S2	Yes	Oblique rotation sensitivity check, omega-squared sensitivity estimates, FDR-adjusted analyses, supplementary ANCOVA, and robustness flags are reported.
Results	13(a)	Report numbers of individuals at each stage of the study.	Section 2.1 Participants	Yes	The manuscript reports approximately 400 parents approached, 320 responses, 25 exclusions, and 295 valid cases.
Results	13(b)	Give reasons for non-participation or exclusions at each stage where applicable.	Section 2.1 Participants	Yes	Exclusions due to incomplete responses, patterned responses, or failure to meet inclusion criteria are reported.
Results	13(c)	Consider use of a flow diagram.	Not included	Not applicable	A flow diagram was not used; participant flow is described narratively.
Results	14(a)	Give characteristics of study participants.	Section 2.1; Table 1	Yes	Participant demographics and child disability characteristics are reported.
Results	14(b)	Indicate numbers of participants with missing data for each variable of interest.	Section 2.1 Participants	Partial	The manuscript reports exclusion of incomplete responses and complete-case analysis but does not provide variable-by-variable missingness because only complete cases were analyzed.
Results	15	Report numbers of outcome events or summary measures.	Section 3; Tables 4–5; Supplementary Table S3	Yes	Means, standard deviations, correlations, subgroup comparisons, p-values, effect sizes, confidence intervals, and subgroup descriptive statistics are reported.
Results	16(a)	Give unadjusted estimates and, if applicable, confounder-adjusted estimates and their precision.	Sections 3.2–3.8; Table 5; Supplementary Table S2	Yes	Primary subgroup comparisons and supplementary ANCOVA results are reported, along with FDR-adjusted p-values, effect sizes, and confidence intervals where applicable.
Results	16(b)	Report category boundaries when continuous variables were categorized.	Section 2.2; Tables 1–2	Yes	Household income, service duration, and monthly treatment cost categories are reported.
Results	16(c)	If relevant, consider translating estimates of relative risk into absolute risk.	Not applicable	Not applicable	The study did not estimate relative risks or odds ratios.
Results	17	Report other analyses, including subgroup and sensitivity analyses.	Sections 3.2–3.8; Table 5; Supplementary Tables S1–S3	Yes	Subgroup comparisons, FDR-adjusted results, supplementary ANCOVA results, CFA/invariance, and descriptive subgroup statistics are reported.
Discussion	18	Summarize key results with reference to study objectives.	Sections 4.1 and 6	Yes	The Discussion and Conclusions summarize subgroup differences and the associative nature of the findings.
Discussion	19	Discuss limitations, considering potential sources of bias or imprecision.	Section 5 Limitations and Future Research	Yes	Limitations related to cross-sectional design, convenience sampling, self-report, omitted covariates, adapted measurement, multiple comparisons, small cell sizes, and service-level characteristics are discussed.
Discussion	20	Give a cautious overall interpretation considering objectives, limitations, multiplicity, related studies, and other evidence.	Sections 4–6	Yes	The manuscript emphasizes preliminary associative evidence and avoids causal or service-specific claims.

Discussion	21	Discuss the generalizability of the study results.	Section 5 Limitations and Future Research	Yes	The manuscript discusses limited generalizability to non-APA users, other rehabilitation modalities, discontinued users, and other countries or service systems.
Other information	22	Give the source of funding and the role of funders.	Funding section	Yes	The manuscript states that the research received no external funding.

Note. This checklist identifies where each STROBE item is addressed in the manuscript. Page and line numbers may change during production; therefore, locations are indicated by manuscript section, table, or supplementary table.
